# Changes in Brain Volume Resulting from Cognitive Intervention by Means of the Feuerstein Instrumental Enrichment Program in Older Adults with Mild Cognitive Impairment (MCI): A Pilot Study

**DOI:** 10.3390/brainsci11121637

**Published:** 2021-12-11

**Authors:** Tzvi Dwolatzky, Refael S. Feuerstein, David Manor, Shlomit Cohen, Haim Devisheim, Michael Inspector, Ayelet Eran, David Tzuriel

**Affiliations:** 1 Rambam Health Care Campus, Haifa 3525433, Israel; da_manor@rambam.health.gov.il (D.M.); minspector212@gmail.com (M.I.); a_eran@rambam.health.gov.il (A.E.); 2 Ruth and Bruce Rappaport Faculty of Medicine, Technion-Israel Institute of Technology, Haifa 3109601, Israel; 3 Feuerstein Institute, Jerusalem 91077, Israel; Rafifu@icelp.org.il (R.S.F.); cohensb@gmail.com (S.C.); haimd@icelp.org.il (H.D.); David.tzuriel@biu.ac.il (D.T.); 4 Faculty of Life Sciences, University of Haifa, Haifa 3498838, Israel; 5 School of Education, Bar-Ilan University, Ramat Gan 5290002, Israel

**Keywords:** brain morphometry, magnetic resonance imaging, cognition, mild cognitive impairment, Feuerstein Instrumental Enrichment, structural cognitive modifiability, mediated learning experience

## Abstract

There is increasing interest in identifying biological and imaging markers for the early detection of neurocognitive decline. In addition, non-pharmacological strategies, including physical exercise and cognitive interventions, may be beneficial for those developing cognitive impairment. The Feuerstein Instrumental Enrichment (FIE) Program is a cognitive intervention based on structural cognitive modifiability and the mediated learning experience (MLE) and aims to promote problem-solving strategies and metacognitive abilities. The FIE program uses a variety of instruments to enhance the cognitive capacity of the individual as a result of mediation. A specific version of the FIE program was developed for the cognitive enhancement of older adults, focusing on strengthening orientation skills, categorization skills, deductive reasoning, and memory. We performed a prospective interventional pilot observational study on older subjects with MCI who participated in 30 mediated FIE sessions (two sessions weekly for 15 weeks). Of the 23 subjects who completed the study, there was a significant improvement in memory on the NeuroTrax cognitive assessment battery. Complete sets of anatomical MRI data for voxel-based morphometry, taken at the beginning and the end of the study, were obtained from 16 participants (mean age 83.5 years). Voxel-based morphometry showed an interesting and unexpected increase in grey matter (GM) in the anterolateral occipital border and the middle cingulate cortex. These initial findings of our pilot study support the design of randomized trials to evaluate the effect of cognitive training using the FIE program on brain volumes and cognitive function.

## 1. Introduction

Aging is often associated with physical and cognitive comorbidity. Deterioration in cognitive function may lead to mild cognitive impairment (MCI) and subsequently to dementia. There is increasing evidence supporting the value of timely recognition and diagnosis of deteriorating cognitive function [1]. Research is now focusing on identifying biological and imaging markers for the early detection of subsequent neurocognitive decline. The discovery of reliable markers will allow for the initiation of pharmacologic and non-pharmacologic interventions that may hopefully delay the onset of cognitive symptoms [2].

MCI is a condition in which individuals develop cognitive impairment with minimal impairment of activities of daily living (IADL) [3]. MCI frequently constitutes a pre-dementia state, particularly for those with amnestic MCI, a condition that may predict the future development of dementia due to the fact of Alzheimer’s disease [4]. The diagnosis of MCI is based on clinical criteria as determined by the Consensus Conference guidelines [3]. The practice guidelines of the American Association of Neurology do not support the use of pharmacological agents for the treatment of MCI. However, non-pharmacological strategies, such as physical exercise and cognitive interventions, are recommended [5,6]. There is increasing interest on the effects of cognitive training on brain plasticity in MCI and early dementia [7].

Cognition-oriented treatments (COTs) are receiving increased interest, both from the scientific community and from the general public [6]. Studies have reported the positive benefits of cognitive training on a number of functions in older adults, including working memory [8]. In fact, a systematic review suggested that the training of executive functions promoted cognitive and neural plasticity in old age [9]. The concept of brain plasticity-based adaptive cognitive training was described in the IMPACT study with positive results on memory and attention [10]. The authors of the IMPACT study report the potential of strategy-based training for promoting brain plasticity. The effect of COTs on brain tissue and function is a fascinating field that should be studied further.

### 1.1. MRI and Cognitive Function

Several MRI techniques are useful as biomarkers of aging and cognitive decline in research and clinical settings [11,12]. Connectivity among cortical regions, probably the most versatile of the MRI biomarkers, is based on functional MRI data. This biomarker has been implemented in studies of the aging human brain [13,14,15,16] as well as for investigating interventions aimed at delaying undesirable aging-related effects [17,18,19]. However, obtaining high-quality functional MRI data depends on the full cooperation of the participants. The requirement of lying still without falling asleep for quite long periods [20] presents a challenge to many older people. In addition, in a longitudinal study design, a high level of cooperation in repeated imaging sessions over time is necessary.

The acquisition of structural MRI is less cumbersome to older participants, since the only requirement is that they adjust to the restrictive environment of the MRI machine and refrain from moving during the scans. Anatomical connectivity can be elegantly studied using diffusion-based MRI, which may detect processes involved in brain aging and dementia [21,22]. There are as yet few reports describing longitudinal follow-up diffusion-based MRI studies in older subjects [23,24]. A possible explanation for the lack of such studies may relate to the fact that reducing scanning times to approximately five minutes, which is tolerable for older populations, requires advanced MRI technology that has only recently become routinely available [25]. Since our MRI system lacked this capability, we utilized morphometric data to measure and map changes in the amounts of brain grey matter (GM), white matter (WM), and cerebrospinal fluid (CSF).

MRI morphometry is gaining acceptance as an adjunct biomarker for differentiating normal aging from the accelerated degeneration of brain tissue associated with various dementias [26,27,28]. Most clinical measurements target specific brain regions, focusing particularly on the hippocampus and entorhinal cortex which are directly implicated in the pathophysiology of common types of dementia [29,30]. Preliminary, comparative evidence shows that an even better correlation with the clinical signs of dementia may be achieved using global mapping of atrophy rates [31]. Research software for implementing global morphometric mapping is now readily available (for example https://www.fil.ion.ucl.ac.uk/spm accessed on 26 October 2021).

Besides these practical considerations, there is also a major substantial incentive to include morphometry in an intervention study. Gradual atrophy of brain tissue is the rule in adults, and this is even more so in the aging brain [12,30,32,33]. Thus, a finding of an increase in the amount of brain tissue would clearly be an exception to the rule, implying a beneficial role for the intervention under investigation.

### 1.2. Study Goals

This study aimed to examine the effect of the mediator-based Feuerstein Instrumental Enrichment (FIE) cognitive training program on the structure of the brain and on cognitive outcomes in older participants with MCI. Our goal was to explore whether this intervention would result in beneficial effects on brain morphometry and cognition.

## 2. Methods

### 2.1. Study Description

We performed a prospective interventional pilot observational study of older subjects with MCI who participated in a period of cognitive training using the FIE program. Measurements included cerebral volumetric MRI and functional MRI imaging to determine the association between this form of cognitive training and changes in imaging metrics. While the functional imaging data are still being processed and analyzed, we report the preliminary findings of the morphometric measurements of the brain over time as well as the cognitive outcomes based on a computerized cognitive assessment battery.

### 2.2. Participants

All participants were residents of the same assisted living center. They were recruited following a lecture explaining the research study. A total of 55 subjects expressed their willingness to be screened for the study and provided their written informed consent. Demographic and medical background data were collected, and a diagnosis of MCI was made based on clinical criteria [3]. A MoCA test (www.mocatest.org accessed on 26 October 2021) was administered for initial cognitive screening, and those who received a score of 18–26 (inclusive), which is compatible with a diagnosis of MCI [34], were included.

We excluded those subjects with a medical or functional condition that would not allow them to participate in the study such as those with an unstable or symptomatic medical condition, depression, bipolar disorder, schizophrenia, or dementia. Subjects with other comorbidities, including cardiovascular disease, cerebrovascular disease, or diabetes, were included if their condition was stable. Those with a MoCA test score of 17 and below or 27 and higher were excluded. Subjects who could not perform an MRI due to the presence of metallic or electronic implants were not included. Based on the criteria outlined above, 23 subjects were considered suitable for inclusion in the study.

### 2.3. Procedure

**Figure 1:**
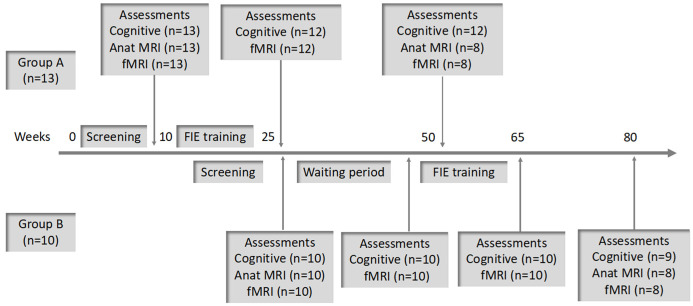
Timeline of study assessments and intervention according to groups. Abbreviations: Anat MRI—Anatomic Magnetic Resonance Imaging; fMRI = Functional MRI; FIE—Feuerstein Instrumental Enrichment.

Since all participants were recruited in a single residential facility, we divided the subjects into two groups (i.e., Group A and Group B). This was done both for administrative reasons (staggering the assessments and periods of intervention) as well as to allow for an extended baseline assessment for functional MRI (two fMRI examinations 15 weeks apart as a waiting period prior to commencement of the intervention in Group B). All participants underwent cognitive and MRI (anatomical and functional) assessments at baseline, cognitive, and functional MRI assessments post-intervention, and cognitive and MRI (anatomical and functional) assessments at one-year post-baseline (Figure 1).

### 2.4. Instruments

#### 2.4.1. The Montreal Cognitive Assessment (MoCA)

The MoCA [34] was developed as a cognitive screening tool for patients with MCI, and it is both sensitive and specific in differentiating mild cognitive decline from preserved cognitive function in older adults. This instrument has been translated and validated for use in Hebrew [35] which was the version used in this study. The test consists of eight sections with a maximum score of 30 points. A score in the range 18–26 is generally considered compatible with a diagnosis of MCI. Those with an educational level of 12 years or less are granted an extra point. The MoCA test is not normalized for age.

#### 2.4.2. CogSym Metacognition Questionnaire

The CogSym instrument comprises 10 questions regarding cognitive symptoms and daily instrumental functional abilities. Subjects are requested to rate their ability on a Likert scale from 1–5 (1 = asymptomatic; 5 = markedly symptomatic) [36]. The symptoms include forgetting names of people in general, forgetting names of close contacts, misplacing belongings, forgetting meetings or events, getting lost in familiar environments, getting confused with days or times, word-finding difficulties, difficulty performing household tasks, difficulty with taking medications, and difficulty with shopping or managing finances. A modified version of this instrument was found to be useful in screening for MCI [37].

#### 2.4.3. Well-Being Questionnaire

Developed by the World Health Organization, this questionnaire comprises 5 items related to subjective well-being [38,39]. The subject is asked to refer to the previous two weeks and indicate for each of the five statements the frequency at which the response is correct on a 5-level scale (5 = all the time, 0 = never). The total raw score, ranging from 0 to 25, is multiplied by 4 to give the final score, with 0 representing the worst well-being and 100 representing the best imaginable well-being.

#### 2.4.4. “NeuroTrax” Computerized Neuropsychological Assessment Battery

This computerized cognitive assessment battery was designed for widespread clinical and research use in evaluating cognitive function (https//www.neurotrax.com accessed on 26 October 2021). The NeuroTrax battery contains standard neurocognitive tests adapted for use in a computerized platform. The tests include verbal and non-verbal memory, Stroop interference, go/no-go, catch game, information processing, visual spatial and verbal functions as well as motor skills [40]. This battery has been shown to significantly discriminate between MCI and cognitively healthy older people across multiple cognitive domains (memory, executive function, visual–spatial skills, language, and attention) and provides a comprehensive profile of global cognitive function [41]. The results are based on norms from a large database and are corrected for age and education level. The mean for each score is 100, and normal values are within 1 standard deviation. Computerized cognitive assessments using the NeuroTrax battery were attained at the pre-waiting period (Group B) and in both groups at the pre-intervention, post-intervention, and follow-up visits.

### 2.5. Cognitive Data Analysis

For the cognitive data analysis, Group A (*n* = 13) was used as the experimental group (pre–post FIE intervention) and Group B (*n* = 10) as a control group (pre–post waiting period) to compare the cognitive changes over time. Statistical analysis was performed by investigating group-by-time interaction effects using repeated measures analysis of variance (ANOVArm) for each cognitive domain and memory component. We also included in the analysis the initial measures (T0) of the different questionnaires (MOCA, the metacognition questionnaire, and the well-being questionnaire) as covariates. We used the IBM^®^ SPSS^®^ software to perform the analysis for the cognitive data.

### 2.6. MRI Data Acquisition and Processing

The experiment consisted of several MRI sessions as detailed in Section 2.3. Complete sets of anatomical MRI data for voxel-based morphometry, taken at the beginning and at the end of the study, were obtained from 16 participants. All MRI sessions took place between 8:00 a.m. and 10:30 a.m. The anatomical scans were performed on a 3T system (GE, MR-750) using a T1-weighted, 3D, inversion-prepared, gradient-echo BRAVO sequence defined with the following parameters: sagittal-oblique orientation, voxel size of 0.9 mm^3^, inversion delay of 450 ms, TR/TE of 6.6/2.5 ms, acceleration factors in the phase/slice directions of 1.75/1.25 for a total time of 3 min and 50 s. An eight-channel head coil was usually used, except for the end-of-the-year session of the first-round group in which, for technical reasons, the head configuration of the clinical HNS coil was employed. To ameliorate the potential bias, we added the group round as a covariate for the statistical model as detailed later.

Preprocessing using the Statistical Parametric Mapping software (SPM12), adapting the optimized protocol described by Good et al. [39], consisted of an initial segmentation of all volumetric images to three constituents: the GM and WM neural tissues and the surrounding CSF. The preprocessing steps that were applied to each tissue segment were: (1) SPM old normalization utility fitting the end-of-the-year image to the beginning-of-the-year image keeping the amounts of the segments in the resulting images; (2) old normalization fitting the beginning-of-the-year to a matching standard MNI template segment and applying the same transformation parameters to the normalized end-of-the-year segment to the written volumes while keeping the concentrations of the segments in the resulting images; (3) spatial smoothing with an 8 mm kernel. The output of this pipeline provided the voxel-wise relative change of each tissue segment over the year.

The final, MNI-space segments were fed into a paired *t*-test factorial design, adding the participant group as a covariate and selecting the ANCOVA regressors in the SPM analysis options. Each tissue segment was analyzed separately. The threshold applied to the statistical maps was *p* < 0.001, uncorrected. The reported GM clusters survived a threshold with *p* < 0.05, corrected for false detection rate.

### 2.7. Intervention

The Feuerstein Instrumental Enrichment (FIE) Program is a cognitive training intervention based on structural cognitive modifiability and the mediated learning experience (MLE) theory [42]. The FIE program aims to promote problem-solving strategies and metacognitive abilities. It focuses on strengthening cognitive functions and on developing learning strategies. MLE processes describe the special quality of interaction between a mediator and a learner. In the MLE interaction, learning is achieved with the active instruction of the mediator who interposes him/herself between the learner and structured stimuli. MLE processes are gradually internalized and integrated by the learner, allowing for modification of cognitive function by means of self-mediation.

The FIE program uses a variety of instruments to enhance the cognitive capacity of the individual as a result of mediation. Each instrument consists of a set of tasks with increasing levels of difficulty, and in a process of active learning, recommendations are provided in order to enable the learner to acquire general principles for domains and contexts beyond the original domains presented. The Feuerstein Institute developed a specific application of the classic FIE designed for cognitive enhancement of aging adults (Feuerstein Memory Program developed by the author RSF). Beyond contributing to the preservation and possible improvement in cognitive function, this program aims to promote feelings of competence and independence and benefits the emotional state.

The classic FIE instruments include the following tasks: organization of dots, comparisons, spatial orientation, analytical perception, and instructions. These tasks are designed to develop skills related to systematic information gathering, reigning in impulsivity, analysis, and planning. The tasks comprising the Feuerstein Memory Program that was used in our study are memory-focused and include the following: processing and memory, the social sphere, everyday functioning, temporal orientation, and figural differences. Emphasis is placed on verbalization and outcome definition. Word fluency is addressed in every session. Strategies are taught to aid retrieval and memory, and focus is placed on strengthening the participants’ orientation skills, categorization abilities, deductive reasoning, and memory.

Specifically, for this study, the Feuerstein Instrumental Enrichment (FIE) Program comprised a combination of classic IE instruments as well as novel instruments. In each session, both classic and novel IE instruments were included in the training program. In our study, FIE was administered in group settings by instructors who were specifically trained to mediate based on the needs of the older population including increased font sizes, larger spaces between the dots, and other adaptations. Subjects participated in 30 mediated FIE sessions (two sessions weekly for 15 weeks, each session continuing for an hour and a half). Attendance data were gathered throughout the intervention period.

## 3. Results

A total of 23 subjects completed the FIE program intervention (13 in Group A and 10 in Group B). The baseline characteristics of the study cohort are presented in Table 1.

### 3.1. Imaging Results

Some of the participants found multiple imaging sessions to be challenging and, therefore, complete, two-session sets of volumetric MRI data for voxel-based morphometry were obtained from only 16 participants, eight participants from each group, five men and 11 women. The mean age was 83.5 (SD 5.0) years. Ten of the participants were widowed, five were married, and one was single.

**Figure 2:**
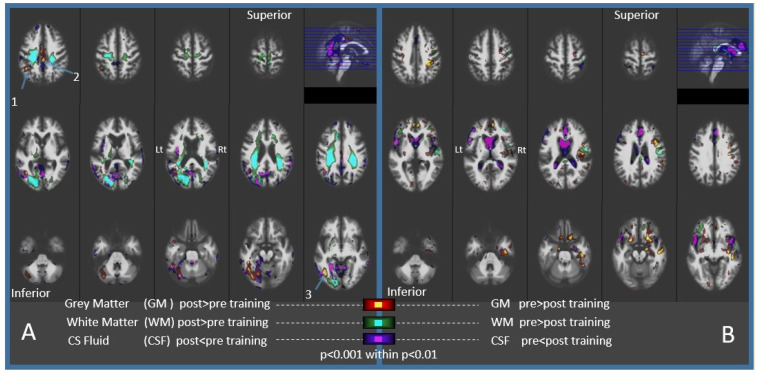
Brain tissue changes over the year that included 15 weeks of the FIE cognitive training. (**A**) Increased GM concentrates in left posterior regions surrounded by decreased CSF. Increased WM extended to the dorsal parts of both hemispheres. Clusters of GM with a higher statistical significance, located on the anterior occipital-lobe border and cingulate cortex are listed in Table 1 (*p* < 0.05, FDR corrected, numbered 1–3). (**B**) Decreased GM was prominent bilaterally in the fronto-basal and in the right temporal regions. Increased CSF was prominent in the lateral ventricles and Sylvian fissures.

Voxel-based morphometry maps of the relative changes in brain tissues over one year in those who participated in cognitive training showed an increase in GM in two regions (Figure 2A and Table 2): (1) The anterolateral occipital lobe, bordering the parietal and temporal lobes of the left cerebral hemisphere. Surrounding the CSF in this region was, correspondingly, decreased: (2) the middle cingulate cortex.

There was an increase in WM, abating the increased GM in the left occipital lobe. But unlike the limited increase in GM, the WM effect was broader, dispersing over the dorsal parts of both hemispheres. Expected, aging-related changes were also observed (Figure 2B). A decrease in GM, ranging from 1.5% to 2.2%, was observed in seven large clusters, encompassing a total volume of 9691 mm^3^, located bilaterally in the frontal-basal regions and diffusely over the right hemisphere. Increased CSF was noticeable in the lateral ventricles as well as in the anterior longitudinal and the Sylvian fissures in both hemispheres.

### 3.2. Cognitive Results

Cognitive data were gathered from a total of 23 subjects including 13 participants from Group A and 10 participants from Group B (Table 3).

In the global cognitive score, there was no significant effect of time (in Group A, pre: M = 91.5, SD = 9.6; post: M = 93.4, SD = 7.0 and in Group B, pre: M = 99.2, SD = 8.8; post: M = 99.5, SD = 7.6, F(1,21) = 0.51, *p* = 0.48), and we did not find any interaction effect (F(1,21) = 0.30, *p* = 0.59).

In memory, there was a significant effect of time with improvement in memory for both groups (in Group A, pre: M = 91.8, SD = 13.5; post: M = 99.7, SD = 9.1; and in Group B, pre: M = 97.6, SD = 9.7; post: M = 99.9, SD = 9.2, F(1,21) = 4.29, *p* = 0.05) with a much larger improvement in Group A than in Group B but without a significant interaction effect (*p* = 0.26).

The significant effect of time was also found in verbal memory (F(1,21) = 9.29, *p* < 0.01), in delayed verbal memory (F(1,21) = 4.40, *p* < 0.05) without interaction effect in verbal memory (F(1,21) = 0.19, *p* = 0.67) and in delayed verbal memory (F(1,21) = 0.21, *p* = 0.66). In non-verbal memory, we did not find any effect of time (F(1,21) = 0.03, *p* = 0.88) and no interaction effect (F(1,21) = 1.44, *p* = 0.25).

In delayed non-verbal memory, we found an interaction effect (F(1,21) = 5.75, *p* < 0.05) with a significant improvement in Group A between pre- and post-intervention (pre: M = 89.8, SD = 11.7, post: M = 97.2, SD = 13.8, *p* < 0.05) whereas no significant difference was found in Group B, apart from a decrease in the delayed non-verbal memory score (pre: M = 105.1, SD = 10.4, post: M = 96.1, SD = 12.6, *p* = 0.12).

In executive function, there was no main effect of time (in Group A, pre: M = 92.0, SD = 11. 4, post: M = 92.2, SD = 8.1; and in Group B, pre: M = 100.6, SD = 11.1, post: M = 101.1, SD = 10.4, F(1,21) = 0.03, *p* = 0.86) and no interaction effect (F(1,21) = 0.01, *p* = 0.93).

In attention there was no main effect of time (in Group A, pre: M = 91.0, SD = 17.9, post: M = 97.5, SD = 11.4; and in Group B, pre: M = 104.5, SD = 13.9, post: M = 103.5, SD = 12.9, F(1,21) = 1.02, *p* = 0.32) and no interaction effect (F(1,21) = 1.95, *p* = 0.18).

In visual spatial abilities, there was no main effect of time (in Group A, pre: M = 91.1, SD = 11.9, post: M = 84.1, SD = 11.8; and in Group B, pre: M = 94.2, SD = 15.2, post: M = 93.5, SD = 16.2, F(1,21) = 3.22, *p* = 0.09) and no interaction effect (F(1,21) = 2.09, *p* = 0.16).

With regard to the MOCA, the metacognition questionnaire and the well-being questionnaire, no effects were found.

## 4. Discussion

Our results indicate that the FIE cognitive training program may induce a sustained, localized increase in GM and WM in older individuals with MCI. This finding is important, since research and clinical protocols that use MRI-based measurements to evaluate brain atrophy in older adults usually demonstrate an association between accelerated brain tissue degeneration and cognitive decline. However, few reports have described an increase in GM over time in adults.

In a previous study, a localized increase in GM was observed, along with functional and anatomical modifications after a period of practicing simultaneous translation [43]. GM and WM volume recovery was observed following abstinence from excessive habitual alcohol consumption [44,45]. The effects in these studies were unrelated to age. There are a couple of reports on increased GM in older adults, which followed months of intense computer game activity [46,47]. One of these studies included participants at risk for MCI [47]. This study measured volume in predefined regions, e.g., the anterior commissural and dorsolateral prefrontal cortices. Another study of patients with subjective memory impairment (SMI), which is considered a possible risk factor for Alzheimer’s disease, showed structural GM volume increases in brain regions encompassing the episodic memory network, with cortical volume expansion of comparable extent as healthy training participants, after two months of episodic memory training. Hippocampal volume increases were detected in the healthy training group but not in the SMI group [48].

Interestingly, most of the previous studies involving the older population included physical activities in their interventions [46,47,49,50]. One of these studies reported an increase in GM in the cingulate gyrus in patients with MCI who participated in physical and cognitive exercise [49]. This is in proximity to the region that we found in our study (Figure 2A, Table 2). A closer look at some of these papers gives the impression that the older population is not usually engaged in the types of activities that have been proven effective in improving cognition, such as dancing [50] or virtual reality gaming [46,47], versus standard sportive activity. The protocol of the current study did not have a physical activity element, but the cognitive approach that was implemented can be considered as a new experience for the participants.

There are few publications that describe GM increase following cognitive intervention alone. One such study applied a default-mode network-based program and found increased GM in healthy older adults [51] but not in MCI patients [52]. They describe a region joining the left cerebellum and the fusiform gyrus across the tentorium. These loci are adjacent to the inferior occipital region detected in our study (Figure 2A, Table 2). The second study [53] engaged amnestic MCI patients in multi-domain cognitive programs which resulted in increased GM in the right hemisphere’s angular gyrus. Our findings include an increase in GM in the left hemisphere’s angular gyrus (Figure 2A, Table 2). Interestingly, the training programs in these studies resemble the multi-faceted approach of the FIE program.

Our morphometric estimations also included the WM, which also increased following the cognitive training. This change was noticed near the foci of increased GM but extended to adjacent regions as well as to the right cerebral hemisphere (Figure 2A). Better characterization of the WM effect may be achieved with diffusion-based MRI measurements.

Behaviorally, the FIE was associated with an improvement in verbal and non-verbal memory. However, an increase in the grey matter was detected in the middle cingulate cortex and not in the retrosplenial cortex-part of the posterior cingulate cortex which is considered to be involved in episodic memory. The midcingulate cortex (MCC_g_) has been proposed to be engaged when predicting and monitoring the outcomes of decisions during social interactions. In particular, the MCC_g_ processes statistical information that tracks the extent to which the outcomes of decisions meet goals when interacting with others [54]. The FIE training took place in a group and was mediated by a trainer and, thus, involved inter-personal interaction. This might explain the increase in the grey matter of the middle cingulate.

We must emphasize that there are a number of limitations to our study. Our imaging results were from a small treatment group of 16 participants. The statistical significance that we found in such a small sample must be regarded with caution, and it is difficult to reliably evaluate the effects of confounding variables such as age and gender. Nevertheless, other reported studies [46,47,50] based their findings on similar numbers of subjects in the main treatment group. Indeed, the statistical significance of our findings accord with those described previously in other studies. Another major limitation of our study is the lack of a proper control group. Taking this into account, there may be two possible explanations for our findings of a localized increase in brain tissue during the follow-up period. On the one hand, the training program may have indeed stimulated those regions of the brain described earlier or, alternatively, the findings may represent a global relative reduction in brain tissue, with the atrophy being more pronounced in certain regions. Being a small pilot study without a control group, we were not able to demonstrate a clear association between cognitive and neural changes. Data from an adequately powered control group would allow for a better understanding of the observed signs of atrophy represented by a decrease in GM and an increase in CSF (Figure 2B) as compared to the effects of the cognitive intervention on brain volume.

## 5. Conclusions

In this pilot study, we report the findings of a localized increase in both cerebral grey matter and white matter in subjects participating in a cognitive intervention program. These findings are in contrast to the expected age-related decrease in brain tissue over time. We also found a significant improvement in memory, particularly in immediate verbal memory and in delayed nonverbal memory. We believe that the results of this small pilot study support further investigation in larger randomized trials to evaluate the effect of cognitive training using the FIE program on brain volumes and cognitive function.

## Figures and Tables

**Table 1:** Characteristics of the study cohort.

	Group A (*n* = 13)	Group B (*n* = 10)	*p*-Values
Sex			
Female	76.9%	80.0%	0.73
Age (years)	82.8	83.7	0.72
Education (years)	11.4	11.6	0.82
MoCA	20.9	22.7	0.06
CogSym	23.3	21.2	0.38
Well-being	18.1	18.6	0.75

Values are presented as the mean. Group A commenced the intervention following baseline assessments. Group B commenced the intervention following a 6 month post-baseline waiting period. MoCA = Montreal Cognitive Assessment; CogSym = CogSym metacognition questionnaire; Well-being = well-being questionnaire.

**Table 2:** Increase in grey matter at one year post-baseline in MCI patients who participated in the FIE Program.

Region	x,y,z (mm) ^a^	Gyri	Cluster (mm^3^)	Effect (%)	T Value	*p* (FDR) ^b^
1	−43, −50, +43	Left Angular	610	2.13	5.34	0.043
		Left Supramarginal				
		Left Inferior Parietal				
2	+2, −27, +40	Left Middle Cingulate	520	1.14	7.97	0.050
		Right Middle Cingulate				
3	−45, −65, −7	Left Inferior Temporal	2850	4.19	7.22	<0.001
		Left Fusiform				
		Left Inferior Occipital				

^a^ Position in the standard MNI (Montreal Neurological Institute) space of the maximum T value in voxel clusters that survived a statistical threshold of uncorrected *p* < 0.001. ^b^ The cluster *p*-values of the false detection rates (FDRs), i.e., odds of discovering such clusters by chance.

**Table 3:** Results for the NeuroTrax cognitive domains and memory sub-components pre-intervention and post-intervention according to groups.

	Group A (*n* = 13)		Group B (*n* = 10)			Repeated Measures ^a^	
**Time**	**Pre**	**Post**	**Pre**	**Post**	**Effect**	**F**	***p*-Value**
Global Score	91.5 (9.6)	93.4 (7.0)	99.2 (8.8)	99.5 (7.6)	Time	0.51	0.48
					Group	4.75	0.04
					Time × Group	0.30	0.59
Memory Domain	91.8 (13.4)	99.7 (9.1)	97.6 (9.7)	99.9 (9.2)	Time	4.29	0.05
					Group	0.64	0.43
					Time × Group	1.32	0.26
Verbal Memory	91.3 (17.8)	104.3 (12.7)	91.3 (15.5)	101.1 (11.4)	Time	9.48	<0.01
					Group	0.10	0.75
					Time × Group	0.19	0.67
Delayed Verbal Memory	93.6 (21.8)	100.6 (13.8)	90.3 (18.9)	101.2 (10.5)	Time	4.40	<0.05
					Group	0.06	0.82
					Time × Group	0.21	0.66
Non-Verbal Memory	92.7 (12.3)	96.5 (15.1)	103.8 (14.6)	100.9 (17.7)	Time	0.03	0.88
					Group	1.95	0.18
					Time × Group	1.44	0.24
Delayed Non-Verbal Memory	89.8 (11.7)	97.2 (13.8)	105.1 (10.4)	96.1 (12.6)	Time	0.05	0.82
					Group	3.37	0.08
					Time × Group	5.75	<0.05
Executive Functions	92.0 (11.4)	92.2 (8.1)	100.6 (11.1)	101.1 (10.4)	Time	0.03	0.86
					Group	5.12	<0.05
					Time × Group	0.01	0.93
Attention	91.0 (17.9)	97.5 (11.4)	104.5 (13.9)	103.5 (12.9)	Time	1.02	0.32
					Group	3.25	0.09
					Time × Group	1.95	0.18
Visual Spatial	91.7 (12.0)	84.1 (11.8)	94.2 (15.2)	93.5 (16.2)	Time	3.22	0.09
					Group	1.37	0.26
					Time × Group	2.09	0.16

Values are presented as the mean (standard deviation). A score of 100 in the NeuroTrax computerized battery is the mean score corrected for age and education, with one standard deviation of 15. ^a^ Group-by-time interaction effects using repeated measures analysis of variance (ANOVArm) for each cognitive domain and memory component.

## Data Availability

All data are available upon request in accordance with confidentiality limitations.
